# Synthesis of anionic ionic liquids@TpBd-(SO_3_)_2_ for the selective adsorption of cationic dyes with superior capacity†

**DOI:** 10.1039/c9ra10035k

**Published:** 2020-02-03

**Authors:** Meng Dang, Qi-Liang Deng, Yan-Yan Tian, Chang Liu, Hai-Peng Shi, Guo-Zhen Fang, Shuo Wang

**Affiliations:** State Key Laboratory of Food Nutrition and Safety, Tianjin University of Science and Technology Tianjin 300457 China fangguozhen@tust.edu.cn s.wang@tust.edu.cn +86-22-60912493 +86-22-60912493; Beijing Advanced Innovation Center for Food Nutrition and Human Health, Beijing Technology & Business University Beijing 100048 China

## Abstract

The discharge of industrial printing and dyeing wastewater is one of the main reasons for the increasing water shortage and deterioration. The treatment of dyestuff wastewater is an issue and needs to be urgently solved. In this work, anionic ionic liquid functional covalent organic materials (COMs) were firstly synthesized and used for the selective adsorption of cationic dyes. First, a series of sulfonic acid group (SO_3_H)-functionalized anionic TpPa-SO_3_, TpBd-(SO_3_)_2_, and TpCR-(SO_3_)_2_ were prepared, respectively, and then imidazole was grafted onto TpBd-(SO_3_)_2_ to obtain ImI@TpBd-(SO_3_)_2_. The full characterization using X-ray diffraction, FT-IR spectroscopy, ^13^C cross-polarization magic-angle spinning NMR spectroscopy, zeta-potentials, BET surface and pore analysis indicated that these COMs and ImI@TpBd-(SO_3_)_2_ exhibited different morphologies, porosities, and potentials. The effects of the type of dye, adsorption time, initial dye concentration, and pH on the adsorption of dyes on ImI@TpBd-(SO_3_)_2_ were systematically investigated, respectively. The results revealed that ImI@TpBd-(SO_3_)_2_ possessed good adsorption performance for nine different cationic dyes with adsorption capacities in the range from 2865.3 mg g^−1^ for methylene blue (MB) to 597.9 mg g^−1^ for basic orange 2 (BO), but little adsorption for anionic and neutral dyes, revealing charge selectivity. The adsorption ratio of ImI@TpBd-(SO_3_)_2_ for MB was as high as 74.0% at 10 min by using 1.0 mg material, owing to the post modification of TpBd-(SO_3_)_2_ with imidazole. The adsorption of MB on ImI@TpBd-(SO_3_)_2_ was pH dependent. The adsorption isotherm and kinetics fitted well with the Freundlich and pseudo second-order kinetic model, respectively. Finally, the very outstanding advantages of superior selective adsorption, desorption, convenient preparation, and low density of ImI@TpBd-(SO_3_)_2_ predicted its research and application potential in dye wastewater recovery.

## Abstract 2

Anionic ionic liquids@covalent organic materials for the selective adsorption of cationic dyes.

## Graphical abstract

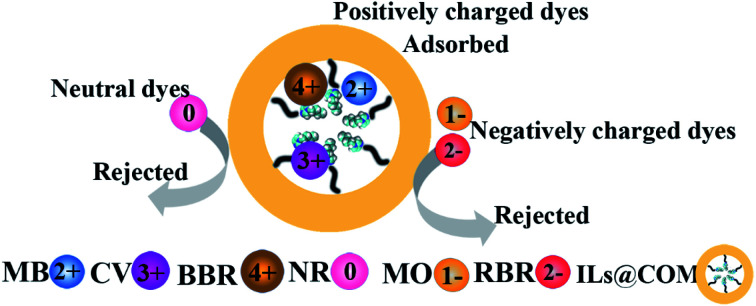

## Introduction

Wastewater caused by the large amount of dye consumption in textile and paper manufacturing industry poses serious threat to mankind and aquatic living organisms because dyes are highly toxic, resistant to degradation, and visible even in trace amounts.^1^ For example, industrial azo dyes, such as Bismarck brown R (BBR) and basic orange 2 (BO) are carcinogenic to humans and poisonous to the environment at low concentrations.^2,3^ Thiazine dyes such as methylene blue (MB) show teratogenic, carcinogenic and mutagenic effects.^4^ Triarylmethane dyes such as crystal violet (CV) have subacute and chronic toxic effects in humans.^5^ To protect the ecological environment and human health, dye discharge is strictly controlled in many countries around the world.^6^ In the pretreatment of high-concentration wastewater, adsorption exhibited great advantages such as simple equipment and convenient operation.^7^ Especially, selective adsorption could not only purify wastewater, but also capture targeted pollutants to recycle them, promoting the transformation of wastewater into resources.^8^ Electrostatic interaction is an excellent candidate for the selective adsorption of targeted dyes. Recently, Wang^9^ synthesized anionic microporous organic polymers (MOPs) to selectively adsorb cationic dyes. Yang^10^ reported poly(ionic liquid)-polyoxometalate hybrids for the selective removal of anionic dyes, proving the function of electrostatic interactions in dye binding. However, these materials possessed shortcomings in practical applications, such as limited capacity, metal ion leakage, limited stability, complicated preparation processes, and poor selective adsorption ability. Hence, developing selective adsorbents with large adsorption capacity synthesized by a simple method is attractive.

In recent years, covalent organic materials (COMs), an exciting new type of porous material, have aroused great interest;^11^ they are constructed by covalent bonds between the organic building units with two and three dimensions. COMs include amorphous porous organic polymers (POPs),^12^ polymers with intrinsic microporosity (PIM),^13^ covalent organic polymers (COPs)^14^ and crystalline ordered covalent organic frameworks (COFs).^15,16^ Due to their unique advantages of various construct structures, functional sites, and diverse pore sizes, COM-based adsorbents are applied to adsorb metal ions,^17^ oil,^18^ bisphenol A,^19^ marine toxins,^20^ and dyes.^21,22^

Ionic liquids (ILs) possess the potential to selectively absorb objects efficiently due to their unique properties of limitless structural tunability and multiple interactions on adsorbate, such as electrostatic interaction, hydrogen bond, van der Waals force, and π–π stacking.^23^ ILs@COMs adsorbent solves the problem of the lack of selectivity to targets of conventional COMs.^24^ In spite of the progress in research on cationic COMs, their complicated preparation procedures or high toxicities of the currently used cationic building units have greatly restricted their practical applications. Besides, the incorporation of anion in the COMs to produce anionic ILs@COMs is a rarely explored area.

**Fig. 1:**
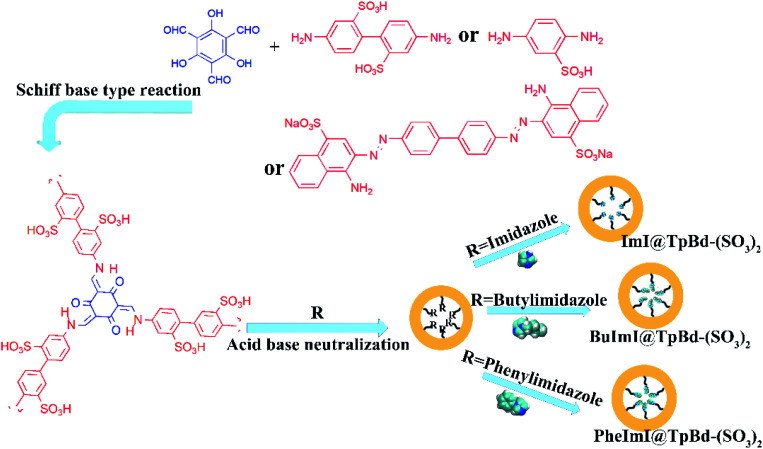
Schematic of TpPa-SO_3_, TpBd-(SO_3_)_2_ and TpCR-(SO_3_)_2_ and anionic ILs@COMs material preparation process.

Herein, we synthesized novel anionic ILs@TpBd-(SO_3_)_2_ with green synthesis method, and used them as adsorbents to selectively adsorb cationic dyes from aqueous solutions (Fig. 1). Their full characterization verified the successful synthesis of these materials. The influential parameters in the adsorption of cationic dye MB on ILs@TpBd-(SO_3_)_2_ include pH, initial concentration, contact time, competitive anionic dyes, and cationic dyes. The adsorption isotherm and kinetic parameter are provided, respectively.

## Experimental

### Chemicals and materials

All chemicals and reagents used were at least of analytical grade. Reagents used in this study are displayed in ESI.†

### Characteristics

Fourier transform-infrared spectra (FT-IR) (4000–400 cm^−1^) in KBr were recorded on a Vector22 spectrometer (Bruker, Germany). The elemental (C, H, N and S) contents of the materials were determined using a Vario MACRO cube Elementar (PerkinElmer, German). Scanning electron microscopy (SEM) images were obtained with a Hitachi SU-1510 (Hitachi, Japan). Field emission transmission electron microscopy (TEM) images were obtained with Jem-2100F (TEM, Jem, Japan). Thermogravimetric analysis (TGA) was carried out using a thermo-gravimetric analyzer (STA449F5, NETZSCH, Germany) at a heating rate of 10 °C min^−1^ up to 800 °C. Powder X-ray diffraction (PXRD) measurement was acquired on a Bruker AXS D8 Advance diffractometer (Bruker, Germany) equipped with Cu Kα radiation (*λ* = 1.5418 Å). ^13^C cross-polarization magic-angle spinning (CP-MAS) was recorded in a Bruker 600 MHz NMR spectrometer. BET surface and pore analysis of the samples were carried out using a Quantochrome Autosorb instrument (Quantatech Co., USA). Zeta potential was measured using a zeta potential analyzer by electrophoresis (Brookhaven ZetaPlus, USA). The electronic analytical balance (model EX125DZH) was obtained from Ohaus International Trading (Changzhou, China). Chromatographic analysis was carried out in an HPLC instrument equipped with a photo-diode array (PDA) (Shimadzu, Japan). HPLC-MS containing a 6410 triple quad mass spectrometry and Agilent 1200 series HPLC instrument was used for HPLC-MS analysis. Multiple positive mode (MRM) was adopted for MS acquisition. The source conditions were as follows: capillary voltage, 4000 V; nebulizer, 40 psi; gas temperature, 350 °C; and gas flow, 10 L min^−1^.

### Synthesis of TpPa-SO_3_, TpBd-(SO_3_)_2_, and TpCR-(SO_3_)_2_

TpPa-SO_3_ was synthesized by a reflux procedure under nitrogen (N_2_) pressure.^25^ Typically, Tp (0.15 mmol) and Pa (0.225 mmol) were dissolved in 25.0 mL ethanol, separately, and then mixed in a 100 mL three-neck round bottom flask equipped with a vacuum link head and N_2_. The flask was vacuumed, and then flushed with N_2_. After stirring for 4 h at 90 °C, the sanguineous suspension TpPa-SO_3_ was obtained. Afterwards, it was washed with DMF and ethanol, respectively, followed by vacuum drying. As for TpBd-(SO_3_)_2_, the reagents Tp (0.075 mmol) and 2,2′-benzidinedisulfonic acid (Bd, 0.1125 mmol) were kept for 8 h at 90 °C.

As for TpCR-(SO_3_)_2_, Tp (0.0.375 mmol) and CR (0.375 mmol) were, separately, dissolved in a mixture of 25.0 mL DMF and mesitylene (v : v 1 : 1). After stirring for 4 h at 105 °C, the black suspension TpCR-(SO_3_)_2_ was obtained, and it was washed with DMF, DMF : mesitylene (v : v) and ethanol, respectively.

### Synthesis of ILs@TpBd-(SO_3_)_2_

Acid–base neutralization is well established for the synthesis of ILs or to introduce functional IL groups in materials.^26^ First, 10.0 mg TpBd-(SO_3_)_2_ was dispersed in ethanol (10.0 mL), which was followed by the addition of 34.0 mg imidazole, 62.1 mg 1-butylimidazole, and 72.1 mg 1-phenylimidazole, separately. The mixture was stirred at room temperature with a rotation speed of 600 rpm. RImI@TpBd-(SO_3_)_2_ was collected by centrifugation and washed with ethanol.

### Adsorption and desorption experiments

The materials (1.0 mg) were immersed in the dye solutions (35.0 mL) and incubated at room temperature for 18 h. For pH adjustment, 1 M HCl or 1 M NaOH was used. After centrifugation, the supernatants were collected respectively, and the responding materials were washed firstly with water and then the eluents, separately. Finally, in single and binary systems, the initial concentration, final concentration in the supernatants, and elution concentration of dyes were analyzed by an UV-vis spectrophotometer, respectively. In the adsorption experiment of a nine-component system, the initial concentration and final concentration of the dyes in the supernatants were analyzed by HPLC-MS, except that of BBR, which was analysed by HPLC. The cationic dye molecules and their properties used in the HPLC-MS analysis including ion pair, fragment ion, and collision energy are displayed in Table S1.†

The adsorbed amounts *Q* (mg g^−1^), elution efficiency of dyes *E* (% the desorption percent of dyes from the materials), and separation efficiency *S* (% the adsorption percent of basic dyes of the total dyes adsorbed on the materials) were calculated on the basis of the following formula:^27,28^1
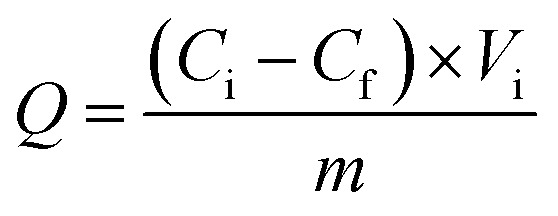
2
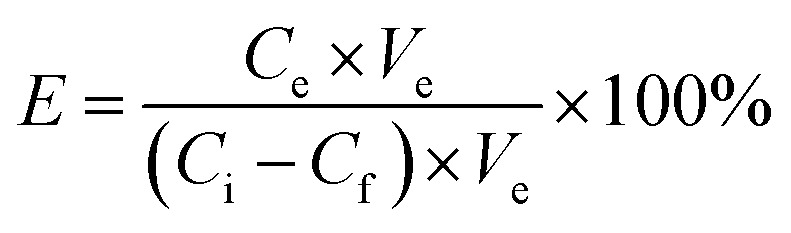

3 *S* = *Q*_MB_/(*Q*_MB_ + *Q*_RBR_) × 100%

in which *C*_i_ and *C*_f_ (mg L^−1^) are the dye concentrations before adsorption and after adsorption, respectively. *C*_e_ (mg L^−1^) is the elution concentration of dyes. *V*_i_ and *V*_e_ (L) are the volumes of the adsorption solution and elution solution, respectively. *M* (g) is the quality of dry material.

#### UV-vis analysis

The maximum absorption wavelengths of the dyes were 533 nm for reactive brilliant red K-2BP (RBR), 464 nm for methyl orange (MO), 663 nm for methylene blue (MB), 451 nm for basic orange 2 (BO), 430 nm for auramine O (AO), 460 nm for Bismarck brown R (BBR), 657 nm for Azure A (AZA), 607 nm for Azure B (AZB), 611 nm for Azure C (AZC), 530 nm for basic red 5 (BR), and 581 nm for crystal violet (CV), respectively.

#### HPLC analysis

The analytes were separated using a C_18_ column (5 μm i.d., 4.6 × 250 mm, Shimadzu, Japan) at a flow rate of 0.8 mL min^−1^ at 30 °C, with the PDA detector at the wavelength of 451 nm and the injection volume of 50 μL. The mobile phases were 0.1% formic acid in water (A), and ACN (B), respectively. The initial gradient conditions were set as 0–12 min, 20–85% B, 13–25 min, 85% B, 25–26 min, 85–20% B, 27–35 min, 20% B.

#### HPLC-MS analysis

The dyes were separated on a ZORBAX XDB-C_18_ column (3.5 μm i.d., 2.1 mm × 150 mm, Agilent, USA). The mobile phases were 5 mmol L^−1^ ammonium acetate and 0.2% formic acid in water (A), and ACN (B), respectively. The initial gradient conditions were set as 0–8.2 min, 20% B with a flow rate of 0.2 mL min^−1^; 8.2–15.0 min, 20–60% B with a flow rate of 0.1 mL min^−1^; 15.0–18.0 min, 60–85% B with a flow rate of 0.2 mL min^−1^; 18.0–19.0 min, 85–20% B with a flow rate of 0.2 mL min^−1^; 20–21 min, 20–20% B, with a flow rate of 0.2 mL min^−1^; the injection volume was 3 μL.

### Adsorption isotherms

The material (1.0 mg) was immersed in the dye solution (35.0 mL) with the initial concentrations was varied from 20 to 130 mg L^−1^, respectively, and agitated at 180 rpm for 18 h at room temperature. Then, 2.0 mL dye solution was pipetted to measure the dye concentration and uptake. The Langmuir and Freundlich isotherm model were applied to fit the experimental data, respectively.^27,28^ The Langmuir isotherm model could be expressed by formula (4)4
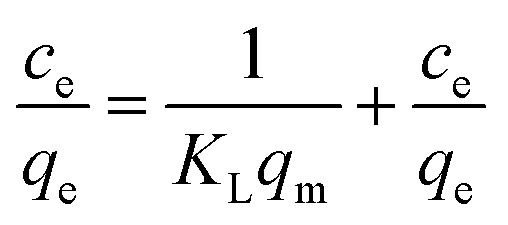


The Freundlich isotherm model adsorption equation is represented as.

5 ln *q*_e_ = *b*_F_ ln *c*_e_ + ln *K*_F_

6 *q*_m_ = *K*_F_ × *c*_e_^*b*_F_^

where *c*_e_ (mg L^−1^) and *q*_e_ (mg g^−1^) are the equilibrium concentration of MB and equilibrium adsorption capacity, respectively. *K*_L_ (L mg^−1^) is the Langmuir constant. *K*_F_ (mg g^−1^) and *b*_F_ are the Freundlich isotherm constants.

### Adsorption kinetics

The material (1.0 mg) was immersed in the dye solution (100 mg L^−1^, 35.0 mL) and agitated at 180 rpm at room temperature. Then 2.0 mL dye solution was pipetted at different time intervals ranging from 10 min to 18 h to measure the dye concentration and uptake. Pseudo-first-order and pseudo-second-order model were utilized to fit the adsorption data, respectively, and they could be described by formula (7) and (8)7
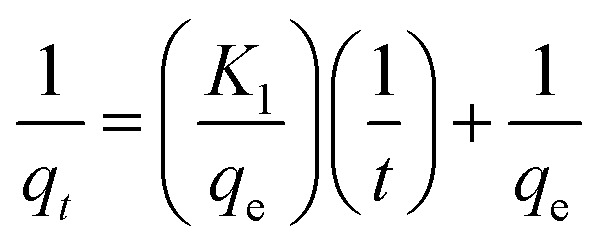
8
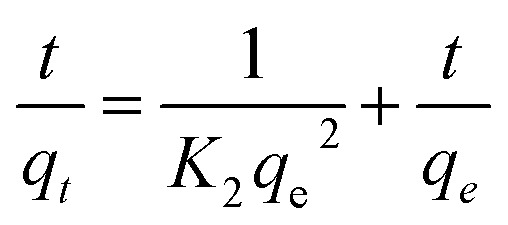
where *q*_*t*_ and *q*_e_ (mg g^−1^) are the adsorption capacities of the dye at time *t* and at equilibrium, respectively. *K*_1_ (min^−1^) and *K*_2_ (g mg^−1^ min^−1^) are the rate constants of pseudo-first-order and pseudo-second-order model, respectively.

### Recyclability test

ImI@TpBd-(SO_3_)_2_ (1.0 mg) was incubated with the dye solution (2.0 mL) for 10 min. After centrifugation, the supernatant was collected and the responding material was washed firstly with water and then the eluent of methanol + 2 mmol L^−1^ ammonium acetate + 0.1% formic acid (95 : 5, v : v). The same operation was repeated five times. The adsorbed amount *Q* (mg g^−1^) was calculated based on formula (1).

### Calculation and simulations

The Forcite module and Pawley refinement of Material Studio 8.0 were employed to simulate the PXRD patterns for crystalline TpPa-SO_3_. First, the structure of TpPa-SO_3_ was constructed assuming *P*1 space groups, respectively. Second, Forcite module including geometry optimization and force field was used for parameterizing the molecular models. Third, PXRD patterns were refined by the Pawley method.

Stable molecular structures of Bd, CR, imidazole, 1-phenylimidazole, 1-butylimidazole, and nine kinds of cationic dyes were calculated, respectively, using Gaussian 09 software under density functional theory (DFT).^29^ Then, the structures were calculated by Multiwfn and displayed through visual molecular dynamics (VMD) in van der Waals (VDW) styles.^30^

## Results and discussion

### Characterization of anionic COMs and ILs@TpBd-(SO_3_)_2_

**Fig. 2:**
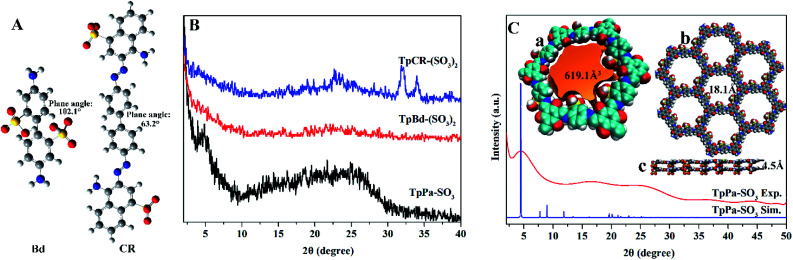
(A) Stable molecular structure of Bd and CR with the angle 102.1° and 63.2° between two planes, respectively. (B) Experimental PXRD profiles of TpPa-SO_3_, TpBd-(SO_3_)_2_, and TpCR-(SO_3_)_2_. (C) PXRD patterns of the synthesized TpPa-SO_3_ (red) compared with the AA stacking mode (blue) of TpPa-SO_3_. (a) The inner cavity volume of TpPa-SO_3_ shown in yellow (619.1 Å). (b) Pore size of TpPa-SO_3_ (18.1 Å). (c) SO_3_H located on the side of the crystallographic plane.

The anionic COMs were synthesized by Tp with Pa, Bd, and CR, respectively. To explore the relationship between the linear linker and crystalline structure, the diamine linker building blocks of the different structures of Bd and CR were calculated by Gaussian and displayed by VMD, respectively (Fig. 2A). Bd and CR contained the angles 102.1° and 63.2° between two benzene ring planes, respectively. As shown in Fig. 2B, PXRD pattern of anionic COMs, TpPa-SO_3_ exhibited a weak diffraction signal at the low angle of ∼4.8° corresponding to the (100) reflection plane, while TpBd-(SO_3_)_2_ and TpCR-(SO_3_)_2_ exhibited no diffraction peak in the region (<5°). The results revealed the long-range molecular ordering of TpPa-SO_3_ (ref. 31 and 32) and amorphous structure of TpBd-(SO_3_)_2_ and TpCR-(SO_3_)_2_.^14^ The reason might be that the benzene rings in Bd and CR were not planar, which caused the distortion and interpenetration of network during the formation of the polymers.^33^ All anionic COMs displayed a broad diffraction peak at 22.5–27.5°, ascribed to the π–π stacking of benzene rings between adjacent layers. As shown in Fig. 2C, the crystalline structure of TpPa-SO_3_ was further simulated using Material Studio 8.0, and an AA stacking structure of the *P*1 space group with *a* = 22.8 Å, *b* = 22.77 Å, *c* = 4.52 Å, *α* = 90°, *β* = 90°, and *γ* = 120° resulted in a PXRD pattern that was in accordance with the experimentally observed pattern (Table S2†). The geometric optimization showed that the inner cavity volume shown in yellow was 619.1 Å^3^ (Fig. 2C(a)), pore size was 18.1 Å (Fig. 2C(b)), SO_3_H group was in the side of the plane of the 2D polymer skeleton, and the center-to-center pore distance in the 2D plane was extended to 4.5 Å (Fig. 2C(c)).

**Fig. 3:**
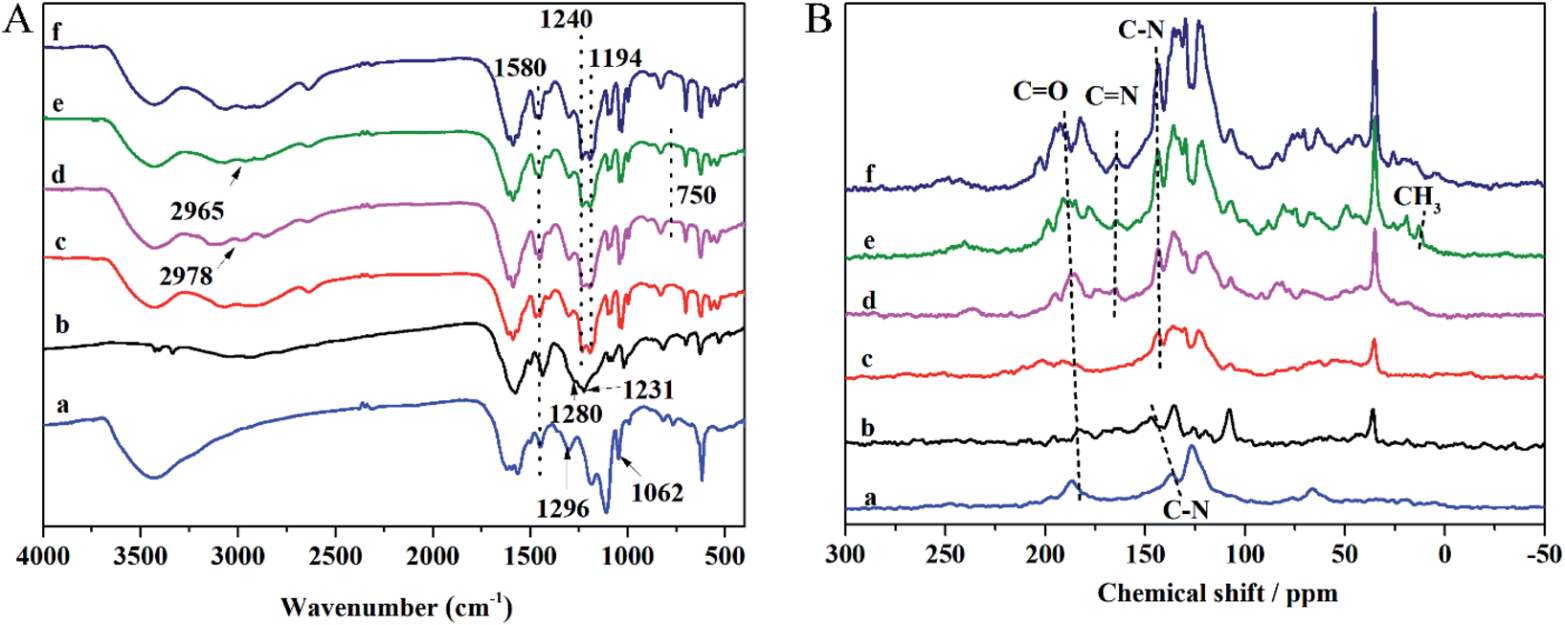
(A) FT-IR spectra and (B) ^13^C CP/MAS NMR spectra of anionic COMs and ILs@TpBd-(SO_3_)_2_; a, b, c, d, e, and f are of TpCR-(SO_3_)_2_, TpPa-SO_3_, TpBd-(SO_3_)_2_, ImI@TpBd-(SO_3_)_2_, BuImI@TpBd-(SO_3_)_2_, and PheImI@TpBd-(SO_3_)_2_, respectively.

The FT-IR and NMR spectra can be applied to analyze the functional groups of materials and determine whether the modification is successful. The FT-IR spectra of anionic COMs and ILs@TpBd-(SO_3_)_2_ are presented in Fig. 3A. TpPa-SO_3_, TpBd-(SO_3_)_2_, and TpBd-(SO_3_)_2_ exhibited bands at 1240, 1280, and 1296 cm^−1^, respectively, which were assigned to C–N stretching vibration. The bands at about 1580 cm^−1^ were attributed to C=C stretching vibration.^31,33^ The bands at 1231, 1194, and 1062 cm^−1^ were owing to SO_2_ stretching vibration.^34,35^ Compared with that in TpBd-(SO_3_)_2_, the new bands at 2978–2965 and 750 cm^−1^ in ImI@TpBd-(SO_3_)_2_ and BuImI@TpBd-(SO_3_)_2_ corresponded to imidazole C–H stretching vibration and imidazole C–H out-plane flexural vibration, respectively.^36^

The ^13^C CP/MAS NMR spectra of the materials are presented in Fig. 3B; all anionic COMs clearly indicated the C–N signal at about 150 ppm and C=O signal at about 180 ppm, indicating the existence of the keto form.^31^ The new bands at about 164 ppm in ILs@TpBd-(SO_3_)_2_ can be attributed to C=N in imidazole, indicating the successful modification of TpBd-(SO_3_)_2_ using imidazole.^25^ BuImI@TpBd-(SO_3_)_2_ possessed an extra peak at 13.0 ppm ascribed to the CH_3_ carbon atoms.

**Fig. 4:**
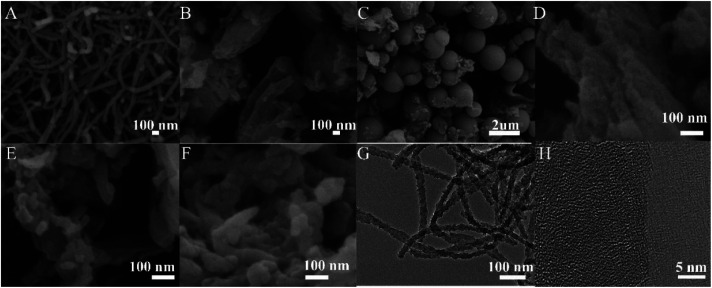
(A), (B), (C), (D), (E) and (F) Represent the SEM images of TpPa-SO_3_, TpBd-(SO_3_)_2_, TpCR-(SO_3_)_2_, ImI@TpBd-(SO_3_)_2_, BuImI@TpBd-(SO_3_)_2_, and PheImI@TpBd-(SO_3_)_2_, respectively. (G) and (H) Represent the HR-TEM images of TpPa-SO_3_.

SEM and HR-TEM were used to observe the surface morphology of anionic COMs and ILs@TpBd-(SO_3_)_2_, and the results are presented in Fig. 4. TpPa-SO_3_, TpBd-(SO_3_)_2_, and TpCR-(SO_3_)_2_ displayed different morphologies, indicating that linear diamines with different lengths had an obvious effect on the morphology. TpPa-SO_3_ was composed of uniform nanofibers with widths of about 20 nm and lengths in the range of 100 nm to tens of micrometres. In the HR-TEM image, some fringes were observed. TpCR-(SO_3_)_2_ was a mixture of micrometre particles and nano-particles, which might be caused by phase separation. TpBd-(SO_3_)_2_ and ILs@TpBd-(SO_3_)_2_ were composed of micrometre rods.

**Fig. 5:**
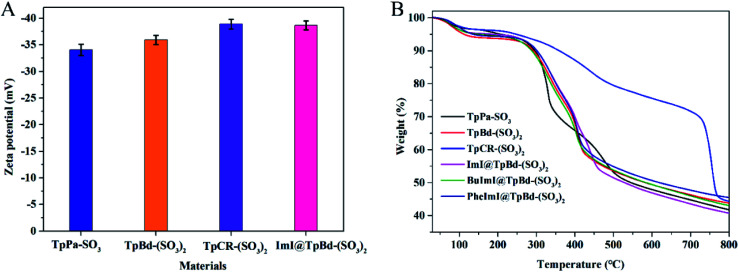
(A) Zeta-potential of TpPa-SO_3_, TpBd-(SO_3_)_2_, and TpCR-(SO_3_)_2_, and ImI@TpBd-(SO_3_)_2_ at pH 7.0. (B) TGA of TpPa-SO_3_, TpBd-(SO_3_)_2_, and TpCR-(SO_3_)_2_ and different ILs@TpBd-(SO_3_)_2_ at 77 K and one bar pressure under N_2_ atmosphere.

As presented in Table S3,† ILs@TpBd-(SO_3_)_2_ and TpBd-(SO_3_)_2_ indicated different C, N, H, and S contents, which confirmed the successful modification of TpBd-(SO_3_)_2_ with ILs. The zeta-potential values of anionic COMs and ImI@TpBd-(SO_3_)_2_ are shown in Fig. 5A. At pH 7.0, disassociation of SO_3_H made the materials negatively charged, which was beneficial to interact with positively charged guests through electrostatic interaction.^37^ The TGA curves of the materials are compared in Fig. 5B. All materials displayed good thermal stabilities upon heating in N_2_ up to 260 °C.

N_2_ adsorption–desorption experiments of COMs and ILs@TpBd-(SO_3_)_2_ were conducted at 77 K and one bar pressure. The samples were activated by degasifying them at 150 °C under vacuum for 8 h. The surface area, pore volume and pore size are shown in Table S4.† TpPa-SO_3_ possessed both micropores and mesopores with pore sizes of 1.7 and 2.9 nm, respectively, while TpBd-(SO_3_)_2_ and TpCR-(SO_3_)_2_ predominantly possessed mesopores of 9.3 and 2.9 nm, respectively. The pore size of TpPa-SO_3_ (1.7 nm) was close to the theoretical value provided by the refinement of the PXRD simulation (Fig. 2C(c)). In crystalline COFs, with the increase in the length of linkers, pore size increased, and pore size could be accurately controlled by delicately designing the length of the building blocks.^15,16^ However, different from this phenomenon, with the increase in the length of linkers (Pa < Bd < CR), pore size changed irregularly, which was probably due to the amorphous structure of TpBd-(SO_3_)_2_ and TpCR-(SO_3_)_2_. The surface area and pore volume of TpPa-SO_3_ and TpBd-(SO_3_)_2_ were 70.8 m^2^ g^−1^, 0.1709 cm^3^ g^−1^ and 60.5 m^2^ g^−1^, 0.1711 cm^3^ g^−1^, respectively, which were much higher than that of TpCR-(SO_3_)_2_ (9.6 m^2^ g^−1^ and 0.0190 cm^3^ g^−1^). SO_3_H-functionalized COMs displayed lower BET surface area and pore volume in comparison with the previously reported TpPa and TpBd series, partly because of the introduction of bulky SO_3_H groups in the pore.^32^ The sizes of imidazole, 1-butylimidazole, and 1-phenylimidazole were smaller than the pore size of TpBd-(SO_3_)_2_ (Fig. S1†), suggesting their potential of entering into the pore during post modification. Notably, the surface area and pore volume of ImI@TpBd-(SO_3_)_2_ have been measured to be 61.8 cm^3^ g^−1^ and 0.1722 nm, respectively, which were almost the same as that of TpBd-(SO_3_)_2_ (60.5 cm^3^ g^−1^ and 0.1711 nm, respectively). However, the pore size of ImI@TpBd-(SO_3_)_2_ was 14.5 and 23.8 nm, which were about 1.6 times that of TpBd-(SO_3_)_2_ (9.3 nm). The reason might be that imidazole changed the arrangement of amorphous TpBd-(SO_3_)_2_. The modification of TpBd-(SO_3_)_2_ with 1-butylimidazole and 1-phenylimidazole obviously decreased the BET surface area and pore volume, probably due to the occupation of these molecules in the pore.

### Adsorption performance of anionic COMs and ILs@TpBd-(SO_3_)_2_ for MB

**Fig. 6:**
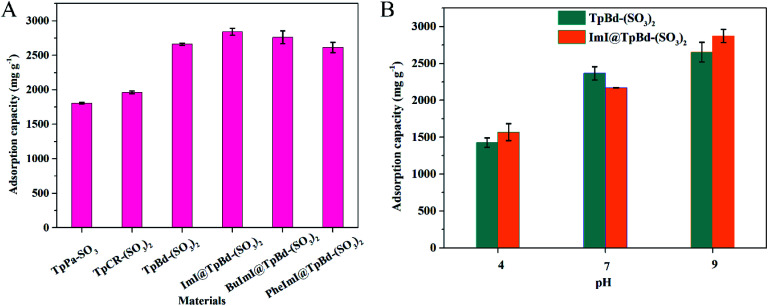
(A) Adsorption performance of MB on COMs and ImI@TpBd-(SO_3_)_2_. Adsorption conditions: *m* = 1.0 mg, *V* = 35.0 mL, *C*_i_ = 100 mg L^−1^ and time 18 h at pH 9.0. (B) Effect of pH on TpBd-(SO_3_)_2_ and ImI@TpBd-(SO_3_)_2_ for MB adsorption. Adsorption conditions: *m* = 1.0 mg, *V* = 35.0 mL, *C*_i_ = 100 mg L^−1^ and time 18 h.

A comparative study of anionic COMs and ILs@TpBd-(SO_3_)_2_ for MB adsorption at pH 9.0 is presented in Fig. 6A. Anionic COMs of TpBd-(SO_3_)_2_ and TpPa-SO_3_ showed the maximum and minimum adsorption capacity (2653.3 and 1806.9 mg g^−1^) for MB, respectively. This obvious difference might be due to the following reasons. On the condition of approximate negative zeta-potential values, although TpPa-SO_3_ showed a greater BET specific surface area, TpPa-SO_3_ displayed the minimum adsorption capacity for MB. In contrast, the pore size of TpBd-(SO_3_)_2_ was five times larger than that of TpPa-SO_3_, and TpBd-(SO_3_)_2_ showed the maximum adsorption capacity for MB, indicating the important effect of mesopores in TpBd-(SO_3_)_2_ in improving the adsorption capacity. The adsorption could be speculated: more free space in the mesopores of TpBd-(SO_3_)_2_ provided the incoming dye molecules a greater area for interaction, accommodated the dyes and facilitated the transport of dyes. In contrast, diffusion was hindered by the narrower pore size of TpPa-SO_3_.^14^ After the modification of TpBd-(SO_3_)_2_ with ILs, all ILs@TpBd-(SO_3_)_2_ displayed outstanding adsorption performance for MB; especially, ImI@TpBd-(SO_3_)_2_ showed the maximum adsorption capacity of 2841.5 mg g^−1^ at pH 9.0, which could compete with any of the other materials reported (Table S5†). The impressive adsorption performance might be attributed to the synergistic effects of pores and densely populated SO_3_H and imidazole functional groups.^37,38^

### Effect of pH

The pH-dependence experiment for MB adsorption on TpBd-(SO_3_)_2_ and ImI@TpBd-(SO_3_)_2_ was carried out by adjusting the pH in the range of 4.0–9.0 with 1 M HCl and 1 M NaOH, respectively. As shown in Fig. 6B, the adsorption performance of TpBd-(SO_3_)_2_ and ImI@TpBd-(SO_3_)_2_ for MB was pH dependent. With pH increasing from 4.0 to 9.0, the adsorption capacity increased, and reached the maximum at pH 9.0. Positively charged MB with N^+^(CH_3_)_3_ group was adsorbed in negatively charged TpBd-(SO_3_)_2_ and ImI@TpBd-(SO_3_)_2_ with SO_3_ group functional through electrostatic interaction. With pH increasing, the adsorption capacity of TpBd-(SO_3_)_2_ and ImI@TpBd-(SO_3_)_2_ for MB increased. The reason might be that the deprotonation of SO_3_ group from the materials increased as pH increased.^39^

### Adsorption equilibrium and adsorption kinetics

**Fig. 7:**
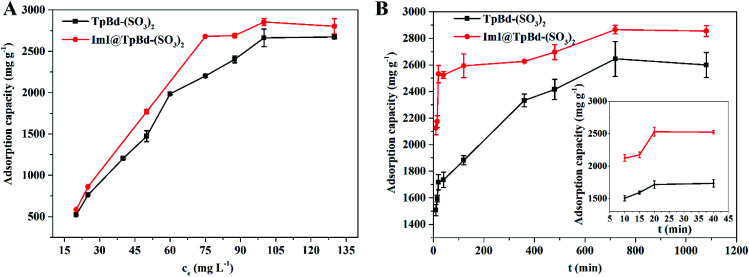
(A) Effects of initial concentration on the MB adsorption property. (B) Effects of contact time on the MB adsorption property. The inset is the adsorption dynamic profiles with time in the range from 10 to 40 min.

Adsorption equilibrium experiment was used to investigate the adsorption mechanism of MB in TpBd-(SO_3_)_2_ and ImI@TpBd-(SO_3_)_2_, respectively. As shown in Fig. 7A, with initial concentrations increasing from 20 to 130 mg L^−1^, the adsorption capacity increased, and the saturation capacity of TpBd-(SO_3_)_2_ and ImI@TpBd-(SO_3_)_2_ was achieved at the MB concentration of 100 mg L^−1^. Fitting the adsorption data using the Langmuir model caused very low correlation coefficients (Fig. S2†). However, as shown in Fig. S3 and Table S6,† the Freundlich isotherm model fit well with the adsorption equilibrium of MB on TpBd-(SO_3_)_2_ and ImI@TpBd-(SO_3_)_2_, with correlation coefficients 0.9496 and 0.9297, respectively. The maximum adsorption capacities of TpBd-(SO_3_)_2_ and ImI@TpBd-(SO_3_)_2_ for MB were calculated to be 2641.2 and 2854.9 mg g^−1^, respectively. These results indicated the multilayer adsorption of MB on heterogeneous surfaces.

Adsorption kinetic experiment was employed to analyse the adsorption rate and adsorption mechanism. As clearly displayed in Fig. 7B, although the adsorption equilibrium was reached at 12 h, the adsorption process was pretty fast in the initial phase. ImI@TpBd-(SO_3_)_2_ (74.0%) revealed a larger adsorption ratio than that of native TpBd-(SO_3_)_2_ (56.9%) at 10 min due to the engineering with imidazole. Besides, ImI@TpBd-(SO_3_)_2_ exhibited a slightly elevated MB adsorption equilibrium capacity (2865.3 mg g^−1^) compared with TpBd-(SO_3_)_2_ (2645.0 mg g^−1^). The fitting plots of the kinetic models and the relative parameters are exhibited in Fig. S4, S5 and Table S7.† The adsorption behavior of MB on TpBd-(SO_3_)_2_ and ImI@TpBd-(SO_3_)_2_ fitted the pseudo-second-order kinetic model very well with *R*^2^ (0.9971 and 0.9988, respectively), indicating that the chemical adsorption of MB on the materials was the rate limiting step. In comparison with TpBd-(SO_3_)_2_, ImI@TpBd-(SO_3_)_2_ further increased the adsorption capacity and adsorption ratio for MB, which could be speculated to be due to the following factors: (i) multiple interactions such as the hydrogen bond and π–π interaction of imidazole counter cation of ImI@TpBd-(SO_3_)_2_ strengthened the interaction with MB;^40,41^ (ii) the post modification of TpBd-(SO_3_)_2_ by imidazole led to the exposure of more favourable adsorption sites and increase in mesopore sizes (Table S4†).

### Charge-selective separation

**Fig. 8:**
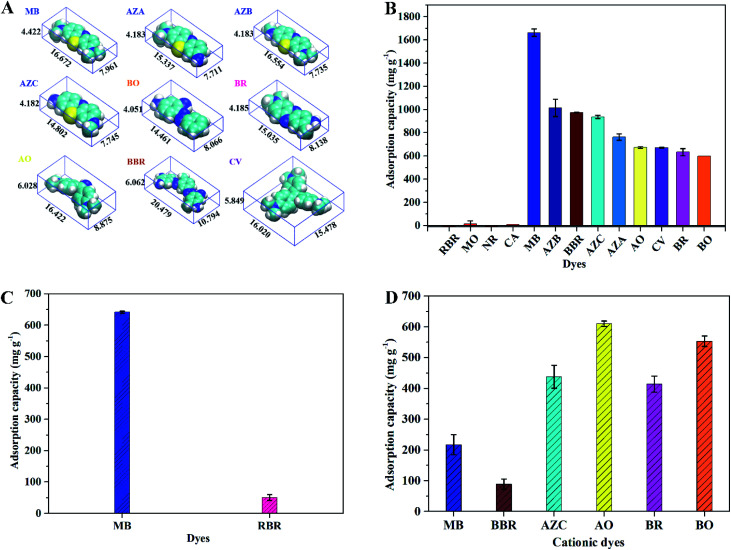
(A) Chemical structures of cationic dye molecules displayed in the VDW style. (B) Single system adsorption performance of ImI@TpBd-(SO_3_)_2_ for anionic, neutral, and cationic dyes. (C) Binary system adsorption performance of ImI@TpBd-(SO_3_)_2_ for MB and RBR. (D) Nine-component system adsorption performance of ImI@TpBd-(SO_3_)_2_ for cationic dyes.

To explore the selective adsorption of charged dyes on ImI@TpBd-(SO_3_)_2_, three kinds of dye molecules with different sizes and charges were chosen as model compounds, including anionic dyes (MO (monoanionic), RBR (dianionic)), neutral dyes (NR, CA), and cationic dyes (MB, AZA, AZB, AZC (dicationic), BO (dicationic), BR (dicationic), AO (tricationic), CV (tricationic), BBR (forcationic)) (Fig. 8A).

### Separation of anionic, neutral, and cationic dyes

Considering the precipitation of some dyes in solution at pH 9.0, the adsorption performance of ImI@TpBd-(SO_3_)_2_ for anionic, neutral, and cationic dye single and mixed systems was evaluated in water at pH 5.5. As shown in Fig. 8B, in single systems, ImI@TpBd-(SO_3_)_2_ indicated high adsorption capacity for all the cationic dyes with different charges and sizes. In contrast, ImI@TpBd-(SO_3_)_2_ showed no adsorption capacity for anionic dyes of small size (MO) and large size (RBR). Also, neither neutral dyes of small size (NR) nor those of large size (CA) were adsorbed. In comparison, ImI@TpBd-(SO_3_)_2_ indicated excellent selective separation performance for cationic dye molecules. The reason was mainly speculated as follows: plenty of SO_3_ groups endowed the material with negative charge on the mesopore walls. In the case that the molecular sizes of all the dyes were lesser than the pore size of ImI@TpBd-(SO_3_)_2_, the difference in the structure of the dye molecules caused different results (Fig. 8A).^27^ Cationic dyes with N^+^ or NH_2_ were adsorbed through electrostatic attraction, while anionic dyes with SO_3_^−^ in the structure were rejected from the materials through electrostatic repulsion. The selectivity was superior to that of the previously reported materials such as graphene oxide and MOFs, which displayed no selective adsorption.^42,43^

Furthermore, the adsorption capacity of ImI@TpBd-(SO_3_)_2_ for cationic dyes was in the range from 1662.6 mg g^−1^ of MB to 597.9 mg g^−1^ of BO at pH 5.5, and decreased in the sequence of MB (1662.6 mg g^−1^) > AZB (1015 mg g^−1^) > BBR (974.1 mg g^−1^) > AZC (936.4 mg g^−1^) > AZA (763.1 mg g^−1^) > AO (672.3 mg g^−1^) > CV (671.1 mg g^−1^) > BR (632.6 mg g^−1^) > BO (597.9 mg g^−1^). Cationic dyes were adsorbed mainly through electrostatic attraction; so, dyes with more positive charges possessed higher adsorption, such as the adsorption capacity of for cationic dyes BBR > tricationic dyes CV > dicationic dyes BR. On the other hand, restricted by the limited specific surface area and pore volume of the material, dyes with a smaller size had a higher adsorption capacity, such as adsorption capacity of dicationic dyes MB > forcationic dyes BBR. The larger BBR molecules might occupy more surface area and pore volume of the material in achieving adsorption saturation, so less BBR was adsorbed. From these results, it could be inferred that the absorption of cationic dyes in ImI@TpBd-(SO_3_)_2_ was mainly driven by electrostatic interaction, and limited by pore volume and specific surface area. The adsorption capacities of ImI@TpBd-(SO_3_)_2_ for MB, CV, and AO were higher than that of most of the reported adsorbents (Table S7†), predicting its potential application in cationic dye removal.

### Separation of anionic and cationic dye mixture

The selective adsorption of specific dyes can not only improve the recovery of dyes but also reduce environmental pollution. So, the separation ability of ImI@TpBd-(SO_3_)_2_ towards dye mixture was also evaluated. To preclude the effect of dye concentration difference, the blue-colored mixture of MB + RBR with a mass ratio of 1 : 1 at an initial pH of 5.5 was treated with ImI@TpBd-(SO_3_)_2_. As shown in Fig. 8C, in a binary system, the adsorption capacity of RBR and MB were 38.0 mg g^−1^ and 641.7 mg g^−1^, respectively. The percent of MB in the MB + RBR mixture adsorbed in ImI@TpBd-(SO_3_)_2_ was 92.7%, indicating the charge-selective separation of the cationic dye from the binary system. The separation mechanism was speculated as follows: in the beginning of the adsorption experiment of the material for the mixture of MB and RBR, the positively charged MB could replace the imidazole counter cations to fit in the channels. Then the MB molecules were immobilized into the pores of ImI@TpBd-(SO_3_)_2_ due to the strong electrostatic interaction between the negatively charged pore walls and cationic MB molecules. A small part of RBR was attracted into ImI@TpBd-(SO_3_)_2_ through electrostatic interaction between MB and RBR. However, although the mesopore could accommodate MB + RBR at the same time, most RBR was rejected from the negatively charged material due to strong electrostatic repulsion.^24^ So the adsorption capacity of ImI@TpBd-(SO_3_)_2_ for RBR slightly increased. The excellent separation of ImI@TpBd-(SO_3_)_2_ for cationic MB from mixtures of MB + RBR was mainly controlled by the electrostatic interaction.

### Discrimination of cationic dyes

As a kind of typical fine industrial products, various cationic dyes are used for industrial dyeing, which has led to the complex composition of cationic dye production wastewater. So, a nine-component system of cationic dyes with different charges and sizes was tested in the research. To exclude the effect of dye concentration difference, the adsorption of cationic dyes on ImI@TpBd-(SO_3_)_2_ was carried out using the nine-component mixture with a mass ratio of 1 : 1. As shown in Fig. 8D, in the nine-component system, ImI@TpBd-(SO_3_)_2_ displayed good adsorption capacities for cationic dyes MB, BBR, AZC, AO, BR, and BO in the range from 88.9 mg g^−1^ of BBR to 610.5 mg g^−1^ of AO. However, ImI@TpBd-(SO_3_)_2_ displayed no adsorption capacities for AZA, AZB and CV. The results clearly showed that the affinity of different dyes to the adsorbed site was different, suggesting a wider application of ImI@TpBd-(SO_3_)_2_ in separation areas such as cationic dyes pre-treatment and chromatographic separation.

### Elution of dye

**Fig. 9:**
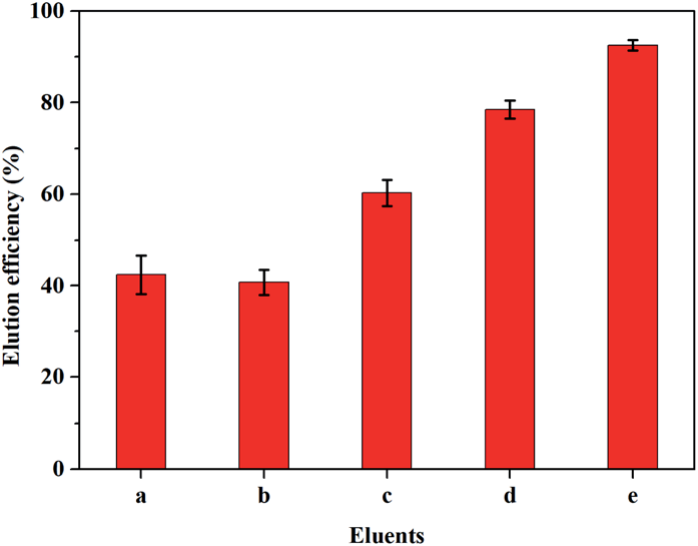
The effects of different eluents on elution efficiency; a = methanol, b = 0.1 mol L^−1^ H_3_PO_4_, c = methanol + 2 mmol L^−1^ ammonium acetate (v : v 95 : 5), d = 0.1 mol L^−1^ SDS, e = methanol + 2 mmol L^−1^ ammonium acetate + 0.1% formic acid (v : v 95 : 5).

Efficient elution of the adsorbed dyes from ImI@TpBd-(SO_3_)_2_ facilitated the recovery of dyes and their application in industry. So, the dye desorption from ImI@TpBd-(SO_3_)_2_ was further established by immersing the samples into six kinds of eluents at 25 °C. As can be seen from Fig. 9, single acid organic solvents or salty solutions failed to make MB desorb from ImI@TpBd-(SO_3_)_2_, while an excellent elution of MB with 92.5% efficiency was achieved by a mixed solution of methanol + 2 mmol L^−1^ ammonium acetate + 0.1% formic acid (95 : 5, v : v).

### Recyclability test

The successive experiments indicated that after five cycles the adsorption capacity remained almost constant (Fig. S6†), which indicated the preferable reusability of ImI@TpBd-(SO_3_)_2_.

## Conclusions

ImI@TpBd-(SO_3_)_2_ was firstly prepared through the convenient modification of TpBd-(SO_3_)_2_ with imidazole, and used for the selective adsorption of cationic dyes of different sizes and charges with superb adsorption capacities. Such factors as the type of material, solution pH, adsorption time, initial dye concentration, and type of dye were systematically investigated to determine the optimal adsorption. As a consequence, the maximum adsorption capacity of ImI@TpBd-(SO_3_)_2_ for MB was 2865.3 mg g^−1^ at pH 9.0. ImI@TpBd-(SO_3_)_2_ displayed super adsorption performance for nine kinds of cationic dyes with maximum adsorption capacity in the range from 1662.6 mg g^−1^ of MB to 597.9 mg g^−1^ for BO at pH 5.5, which was among the highest capacities in cationic dye adsorption. Besides, the study on the adsorption mechanism indicated that the Freundlich model displayed a better fit to adsorption isotherms, and the pseudo-second order model fitted well with adsorption kinetics. Furthermore, ImI@TpBd-(SO_3_)_2_ could selectively adsorb cationic dyes in cationic and anionic binary dye systems, and it showed favorable adsorption performance for nine-component dye mixture. Finally, the convenient preparation, selective adsorption performance, excellent adsorption capacity, desorption, advantages of low density, and very fast adsorption in the initial phase revealed the potential application of ImI@TpBd-(SO_3_)_2_ in dye wastewater separation and recycling.

## Conflicts of interest

There are no conflicts to declare.

## Supplementary Material

RA-010-C9RA10035K-s001
