# Supplementary material for: Synthesis of anionic ionic liquids@TpBd-(SO3)2 for the selective adsorption of cationic dyes with superior capacity
Source: RSC Adv. 2020 Feb 3;10(9):5443–53. doi: 10.1039/c9ra10035k (PMC9049230; doi:10.1039/c9ra10035k)
Supplement: RA-010-C9RA10035K-s001 [file RA-010-C9RA10035K-s001.pdf]

Electronic Supplementary Material (ESI) for RSC Advances.
This journal is © The Royal Society of Chemistry 2020

Supporting Information

Synthesis of anionic Ionic Liquids@Covalent Organic Materials for selective adsorption of cationic dye with superior capacity

Meng Dang[a], Qi-Liang Deng[a], Yan-Yan Tian[a], Chang-Liu[a], Hai-Peng Shi[a], Guo-Zhen Fang[a]*, and Shuo Wang[a,b]*

[a]State Key Laboratory of Food Nutrition and Safety, Ministry of Education, Tianjin University of Science & Technology, Tianjin, 300457, China.

[b]Beijing Advanced Innovation Center for Food Nutrition and Human Health, Beijing Technology & Business University (BTBU), Beijing, 100048, China.

S1. General information

1.Materials and reagents

All chemicals and reagents used were at least of analytical grade. Ultrapure water was prepared in doubly deionized water (DDW, 18.2 MU cm^{-1}) from a Millipore water purication system (Millipore, Billerica, MA, USA). 1,3,5-Triformylphloroglucinol (Tp) was purchased from Yuhao Chemical Technology Co. Ltd. (Hangzhou, China). 2,5-Diaminobenzenesulfonic acid (Pa), and 2,2'-benzidinedisulfonic acid (Bd) were purchased from Macklin Biochemical Co. Ltd. (Shanghai, China). Imidazole, ethanol, methanol, N,N-dimethylformamide (DMF), 1,4-dioxane, mesitylene, phosphorous acid (H_3PO_4), disodium phosphate dodecahydrate ($NaH_2PO_4 \cdot 12H_2O$), sodium dihydrogen phosphate, ($Na_2HPO_4 \cdot 2H_2O$), hydrochloric acid (HCl), sodium hydroxide (NaOH) and acetonitrile (ACN) were purchased from Sinopharm Chemical Reagent Co. Ltd. (Shanghai, China). 1-Phenylimidazole was obtained from TCI (Shanghai, China) and 1-butylimidazole was obtained from Alfa Aesar (Shanghai, China), respectively. Methylene Blue (MB), methyl orange (MO), reactive brilliant red K-2BP (RBR), basic red 5 (BR), crystal violet (CV), and basic orange 2 (BO) were purchased from Sanjiang Chemical Technology Co. Ltd. (Tianjin, China). Nile red (NR) and calcein (CA) were purchased from Yuanye Biotechnology Co. Ltd. (Shanghai, China). Auramine O (AO) was obtained from Adamas Reagent Co. Ltd. (Shanghai, China). Azure A (AZA), Azure B (AZB) and Azure C (AZC) were purchased from Amresco Co. Ltd. (USA). Congo red (CR) and bismarck brown R (BRR) were obtained from J&K Scientific Ltd. (Beijing, China). Arginine was obtained from Sigma-Aldrich (St., Louis, MO).

S2. Figures and Tables

Table S1. Cationic dye molecules and their properties used in HPLC-MS analyze

Analyte	Molecular mass	Parention (m/z)	Fragment ion (m/z) ＊	Cone voltage (V)	Collision energy (eV)	Charge
Methylene Blue	373.9	284.2	268.1	120	40	Positive
			252.2	120	50	
Azure A	291.8	255.8	213.7	130	30	
		255.8	198.8	130	40	
Azure B	305.8	269.8	253.7	130	28	
			227.8	130	30	
Azure C	277.8	241.9	226.8	130	33	
		241.9	199.8	130	26	
Auramine O	303.8	268	147	130	29	
		268	131	130	55	
		268	107	130	33	
Crystal Violet	373.5	372	356.1	130	40	
		372	340	130	51	
Basic Red 5	288.8	253.1	237.1	135	40	
			222	135	50	
			210.1	135	33	
Basic Orange 2	248.7	213.4	120.2	120	15	
			77.2	120	15	

＊ Quantitative ion.

Table S2. Fractional atomic coordinates for the unit cell of TpPa-SO_3

	TpPa-SO_3<br>Space group P 1<br>a = 22.8 Å, b =22.77 Å, c =4.52 Å, α = 90°, β = 90°, γ = 120°		
atom	x	y	z
C	0.01939	0.50187	0.5
C	0.13839	0.5297	0.5
C	0.20854	0.57990	0.5
C	0.23264	0.65104	0.5
C	0.25782	0.55190	0.5
N	0.08742	0.54403	0.5
O	0.19149	0.67203	0.5
O	0.23768	0.49668	0.5
C	0.30249	0.70059	0.5
C	0.32845	0.60643	0.5
C	0.34923	0.6768	0.5
O	0.411162	0.7191	0.5
C	0.32227	0.7707	0.5
N	0.38593	0..82389	0.5
C	0.40856	0.89167	0.5
C	0.37479	0.92767	0.5
C	0.47483	0.92911	0.5
S	0.296207	0.89281	0.5
O	0.29235	0.0.94741	0.5
O	0.28291	0.83109	0.5
O	0.23157	0.86508	0.5
H	0.006561	0.58192	0.5
H	0.10745	0.409597	0.5
H	0.10139	0.59135	0.5
H	0.12483	0.47965	0.5
H	0.28553	0.78279	0.5
H	0.42066	0.81197	0.5
H	0.500075	0.90501	0.5

Table S3 Comparison of element analyses of C, N, H, and S

Materials	C (%)	N (%)	H (%)	S (%)
TpPa-SO_3	42.21	11.58	5.35	13.35
TpBd-$(SO_3)_2$	41.93	8.60	5.15	10.04
TpCR-$(SO_3)_2$	34.67	6.23	3.06	8.05
ImI@TpBd-$(SO_3)_2$	46.59	8.66	6.08	8.85
BuImI@TpBd-$(SO_3)_2$	42.94	8.62	5.69	9.97
PheImI@TpBd-$(SO_3)_2$	43.1	8.28	5.77	10.89

Table S4 Comparison of surface area and pore volume of the as-prepared materials

Materials	Surface area (m^2g^{-1})	Pore volume (cm^3g^{-1})	Pore size (nm)
TpPa-SO_3	70.8	0.1709	1.7, 2.9
TpBd-$(SO_3)_2$	60.5	0.1711	9.3
TpCR-$(SO_3)_2$	9.6	0.0190	2.9
ImI@TpBd-$(SO_3)_2$	61.8	0.1722	14.5, 23.8
BuImI@TpBd-$(SO_3)_2$	33.7	0.1245	2.3, 14, 21
PheImI@TpBd-$(SO_3)_2$	40.2	0.1125	6.8

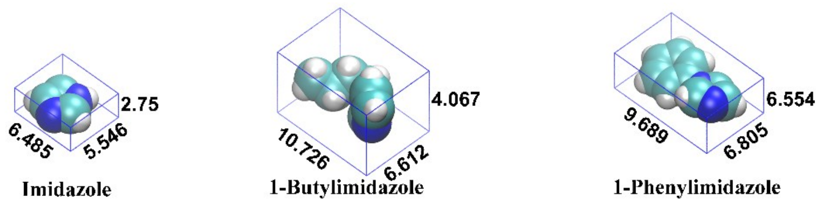

Fig. S1 The molecular models of imidazole derivatives were displayed in the Bondi van der waals (VDW) style (blue, carbon; white, hydrogen; blue, nitrogen) calculated by Multiwfn; Imidazole with a size of 6.485×5.546×2.75 Å, 1-buthylimidazole with a size of 10.726×6.612×4.067 Å, and 1-phenylimidazole with a size of 9.689×6.805×6.554 Å

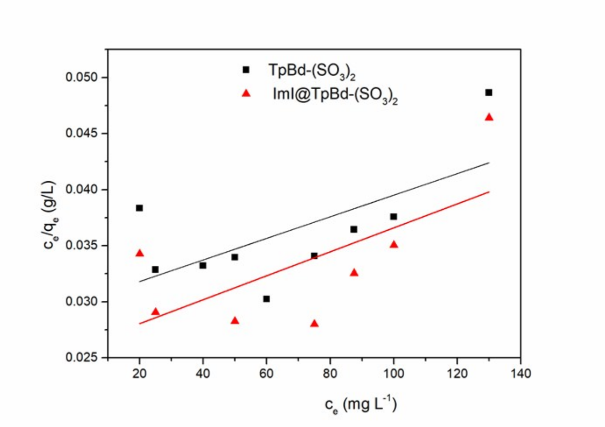

Fig. S2 Langmuir model of MB in TpBd-$(SO_3)_2$ and ImI@TpBd-$(SO_3)_2$

Table S5 Comparison of cationic dyes adsorption

Materials	q_e (mg g^{-1})	Ref
KOH-activated carbon from sucrose	MB 704.2	S1
Sulfuric acid activated (RHS) activated rice husk carbon	CV 64.9	S2
Magnetic nanocomposite beads comprising carboxylic acid functionalized carbon nanotube	MB 465.5; Direct red 380.7	S3
Fe_3O_4@polydopamine-Ag hollow microspheres	MB 102.0	S4
EDTA-Cross-Linked β-Cyclodextrin	MB 88.5; CV 114	S5
Aminocarboxylate/maleic acid resin	MB 2101; Hg(II) 263	S6
Bakelite-type anionic microporous organic polymers	MB 712.2; MG 593.6	S7
Magnetic graphene oxide modified zeolite	MB 97.3	S8
Poly (NIPAAm/AA/N-allylisatin) nanohydrogel	MB 392.2; AO 337.8; BO 961.5	S9
Magnetic polyacrylamide microspheres	MB 1990; GV 1850; BR 1937	S10
ImI@TpBd-$(SO_3)_2$	MB 2865.3; AZB 1015; BBR 974.1; AZC 936.3; AZA, AO, CV, BR, BO 597.9-763.1	This work

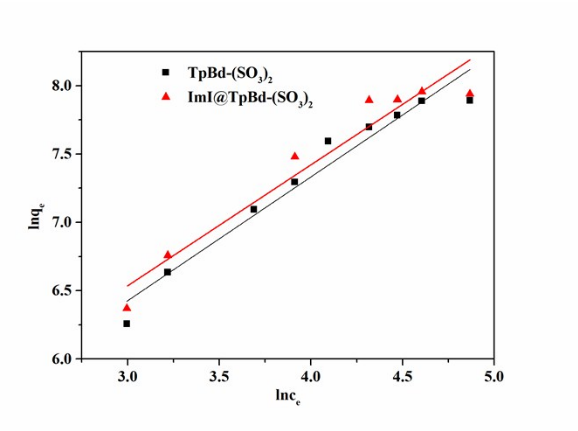

Fig. S3 Freundlich model of MB in TpBd-$(SO_3)_2$ and ImI@TpBd-$(SO_3)_2$

Table S6 Freundlich model constants and correlation coefficient

Materials	Freundlich model			
	K_F (mg g^{-1})	b_F	q_e(cal) (mg g^{-1})	R^2
TpBd-$(SO_3)_2$	40.703	0.906	2641.2	0.9496
ImI@TpBd-$(SO_3)_2$	48.444	0.885	2854.9	0.9297

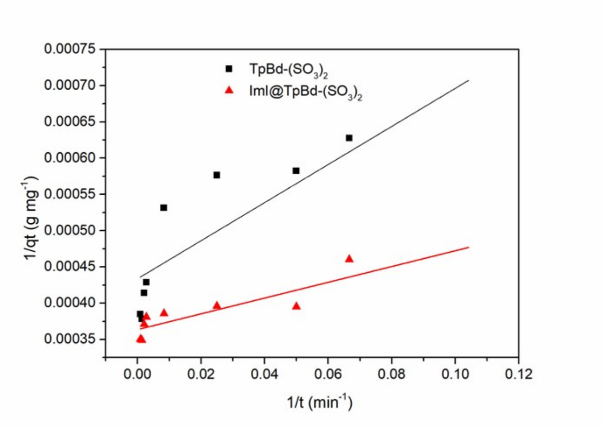

Fig. S4 Pseudo-first-order model of MB in TpBd-$(SO_3)_2$ and ImI@TpBd-$(SO_3)_2$

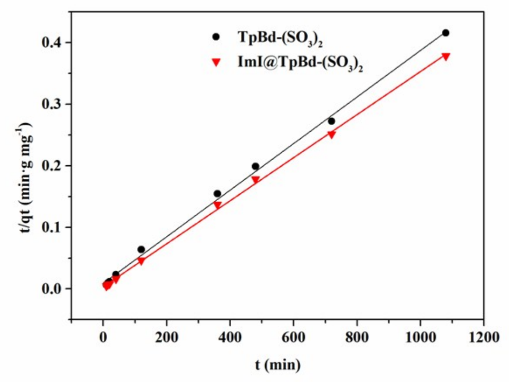

Fig. S5 Pseudo-second-order model of MB in TpBd-$(SO_3)_2$ and ImI@TpBd-$(SO_3)_2$

Table S7 Comparison of kinetic model parameters of TpBd-$(SO_3)_2$ and ImI@TpBd-$(SO_3)_2$

Type	Parameter	TpBd-$(SO_3)_2$	ImI@TpBd-$(SO_3)_2$
Pseudo-first-order kinetics	q_e (mg g^{-1})	2307.4	2748.7
	k_1 (min^{-1})	6.068	2.996
	R^2	0.7492	0.8449
Pseudo-second-order kinetics	$q_{e,exp}$ (mg g^{-1})	2645.0	2873.2
	q_e (mg g^{-1})	2627.0	2866.2
	k_2 (g $mg^{-1}min^{-1}$)	1.6227×10^{-5}	3.493×10^{-5}
	R^2	0.9971	0.9988

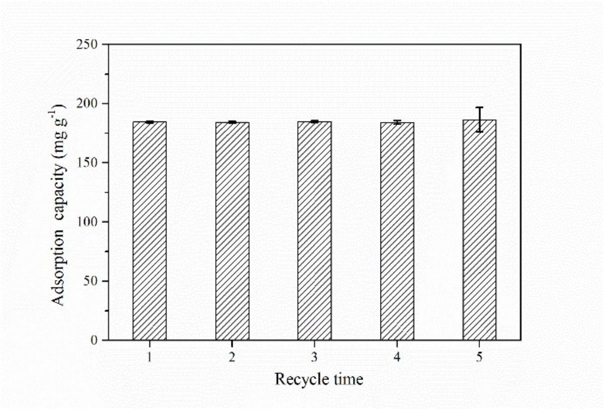

Fig. S6 Recycle studies of MB adsorption in ImI@TpBd-$(SO_3)_2$

S3. References

[1] K. C. Bedin, A. C. Martins, A. L. Cazetta, O. Pezoti, V. C. Almeida, KOH-activated carbon prepared from sucrose spherical carbon: adsorption equilibrium, kinetic and thermodynamic studies for methylene blue removal, Chem. Eng. J. 286 (2016) 476-484.

[2] K. Mohanty, J.T. Naidu, B.C. Meikap, M. N. Biswas, Removal of crystal violet from wastewater by activated carbons prepared from rice husk, Ind. Eng. Chem. Res. 45 (2006) 5165-5171.

[3] S. Saber-Samandari, S Saber-Samandari, H. Joneidi-Yekta, M. Mohseni, Adsorption of anionic and cationic dyes from aqueous solution using gelatin-based magnetic nanocomposite beads comprising carboxylic acid functionalized carbon nanotube, Chem. Eng. J. 308 (2017) 1133-1144.

[4] K. Cui, B. Yan, Y. Xie, H. Qian, X. Wang, Q. Huang, Y. He, S. Jin, H. Zeng, Regenerable urchin-like Fe_3O_4@PDA-Ag hollow microspheres as catalyst and adsorbent for enhanced removal of organic dyes, J. Hazard. Mater. 350 (2018) 66-75.

[5] F. Zhao, E. Repo, D. Yin, Y. Meng, S. Jafari, M. Sillanpää, EDTA-Cross-Linked β-Cyclodextrin: An Environmentally Friendly Bifunctional Adsorbent for Simultaneous Adsorption of Metals and Cationic Dyes, Environ. Sci. Technol. 49 (2015) 10570-10580.

[6] S.A. Ali, I.Y. Yaagoob, M.A.J. Mazumder, H. A. Al-Muallem, Fast removal of methylene blue and Hg(II) from aqueous solution using a novel super-adsorbent containing residues of glycine and maleic acid, J. Hazard. Mater. 369 (2019) 642-654.

[7] B Wang, Q Zhang, G Xiong, F Ding, Y He, B Ren, L. You, X. Fan, C. Hardacre, Y. Sun, Bakelite-type anionic microporous organic polymers with high capacity

for selective adsorption of cationic dyes from water, Chem. Eng. J. 366 (2019) 404-414.

[8] T. Huang, M. Yan, K. He, Z. Huang, G. Zeng, A. Chen, M. Peng, H. Li, L. Yuan, G. Chen, Efficient removal of methylene blue from aqueous solutions using magnetic graphene oxide modified zeolite, J. Colloid Interface Sci. 543 (2019) 43-51.

[9] Viran P. Mahida, Manish P. Patel, Removal of some most hazardous cationic dyes using novel poly (NIPAAm/AA/N-allylisatin) nanohydrogel, Arab. J. Chem. (2016) 9 430-442.

[10] T. Yao, S. Guo, C. Zeng, C. Wang, L. Zhang, Investigation on efficient adsorption of cationic dyes on porous magnetic polyacrylamide microspheres, J. Hazard. Mater. 292 (2015) 90-97.
